# Nanovaccines for lung cancer: Platforms, mechanistic insights, and translational challenges

**DOI:** 10.1016/j.pccm.2026.02.001

**Published:** 2026-03-10

**Authors:** Hamed Hosseinalizadeh, Fujing Ge, Mohsen Davari, Xiaohong Liu, Lei Tian, Jianhua Yu

**Affiliations:** a Faculty of Paramedicine, Guilan University of Medical Sciences, Rasht 41937-1311, Iran; b Division of Hematology/Oncology, Department of Medicine, School of Medicine, University of California, Irvine, CA 92697, USA; c Faculty of Medicine, Hormozgan University of Medical Sciences, Bandar Abbas 79199-15519, Iran; d Comprehensive Cancer Center, City of Hope, Los Angeles, CA 91010, USA; e Chao Family Comprehensive Cancer Center, University of California, Irvine, CA 92697, USA

**Keywords:** Lung cancer, Nanovaccine, Neoantigen, Combination therapy, Tumor antigen

## Abstract

Lung cancer remains a global health challenge, with high mortality rates and frequent resistance to conventional therapies. In recent years, nanovaccines-based immunotherapy has emerged as a promising frontier, harnessing advances in nanotechnology to enable precise delivery of tumor-associated antigens, potentiate immune activation, and overcome barriers imposed by the tumor microenvironment. This review synthesizes current knowledge and recent progress in the field, highlighting several nanovaccine platforms from mRNA and protein constructs to biomimetic designs, which have demonstrated preclinical efficacy against both primary and metastatic lung cancer. We illustrate how innovations can elicit robust polyclonal T cell responses and remodel the immune landscape within tumors, including co-delivery of antigens and adjuvants, use of patient-derived materials, and integration of multi-omics. Furthermore, the combination of nanovaccines with current therapies, including immune checkpoint inhibitors, cell-based therapies, and gene-modulating strategies, offers new avenues for enhancing therapeutic outcomes. Despite these advances, several challenges remain, including barriers of large-scale manufacturing, safety assessment, regulatory approval, and the need for personalized optimization. Ongoing research and clinical studies will be essential to translate these experimental successes into personalized treatments for patients with lung cancer. Nanovaccine immunotherapy stands poised to become a transformative component in the evolving landscape of lung cancer management.

## Introduction

Lung cancer remains the leading cause of cancer-related mortality worldwide, accounting for approximately 1.8 million deaths each year.^1^, ^2^, ^3^, ^4^ Non-small cell lung cancer (NSCLC) comprises about 85% of all cases, and most patients are diagnosed at advanced stages, resulting in a dismal 5 year survival rate below 20%.^5^^,^^6^ Despite significant advances in early detection and molecular diagnostics, lung cancer is characterized by rapid progression, high rates of metastasis (particularly to the brain, liver, and bones), and frequent recurrence after initial treatment.^3^^,^^7^ The complex tumor microenvironment (TME), dominated by immune-suppressive cells such as tumor-associated macrophages (TAMs) and regulatory T cells, further hampers effective anti-tumor responses.^8^^,^^9^ As a result, overcoming immune evasion and metastatic dissemination remains a formidable challenge in the management of lung cancer.

Conventional therapies, including surgery, chemotherapy, and radiotherapy, offer limited long-term benefit for most lung cancer patients, particularly those with advanced or metastatic disease.^10^, ^11^, ^12^ Chemotherapy is linked to considerable toxicity, immunosuppression, and non-specific cytotoxic effects,^13^ while radiotherapy is limited by normal tissue damage and reduced efficacy against micrometastatic disease.^14^ Although targeted therapies and immune checkpoint inhibitors have revolutionized the therapeutic landscape, only a small fraction of patients derives durable benefit, hindered by the development of resistance, tumor heterogeneity, and immune escape.^15^, ^16^, ^17^ Innovative immunotherapy approaches are urgently needed which could generate robust, durable, and tumor-specific immune responses.

Nanovaccines (NVs) have emerged as a transformative approach in cancer immunotherapy, offering multiple advantages over conventional vaccines and therapies.^18^ By nanoscale carriers, such as liposomes, biomimetic vesicles, and polymeric nanoparticles (NPs), NVs efficiently deliver tumor-associated antigens (TAAs) along with potent adjuvants to antigen-presenting cells, thereby promoting strong cytotoxic T cell responses and immune memory.^19^ Several innovative platforms have been developed, including messenger RNA (mRNA)-based NVs, recombinant protein or peptide vaccines, cell membrane-coated NPs, and autologous NVs derived from tumor tissues.^19^ Mechanistically, NVs can modulate the TME, reprogramming TAMs, activating migratory dendritic cells (DCs), and inducing immunogenic cell death (ICD) and long-lasting memory T cells.^20^ Notably, recent studies have demonstrated that NVs elicit cross-protective immune responses, prevent metastasis and recurrence, and synergize with immune checkpoint inhibitors to convert “cold” into “hot” immune-infiltrated tumors.^21^ Collectively, these features highlight the unique potential of NVs as next-generation immunotherapeutics for lung cancer, capable of overcoming the barriers that limit current treatments and opening new avenues toward durable remission and improved survival.

In this review, we aim to provide a comprehensive summary of NVs in the prevention, treatment, and immunomodulation of lung cancer. Drawing on the latest advances from preclinical and clinical research, including NV platforms, mechanistic innovations, and translational strategies, we critically summarize the immunological foundations, technological developments, and therapeutic potential of NVs for lung cancer. By integrating evidence from recent studies, we discuss how NVs overcome the limitations of current therapies, modulate the TME, and induce durable anti-tumor immune responses. Finally, we highlight the key challenges and future directions that needed to be addressed to translate these promising technologies into effective clinical solutions for patients with lung cancer.

## Immunological and biological basis of NVs

**Fig. 1:**
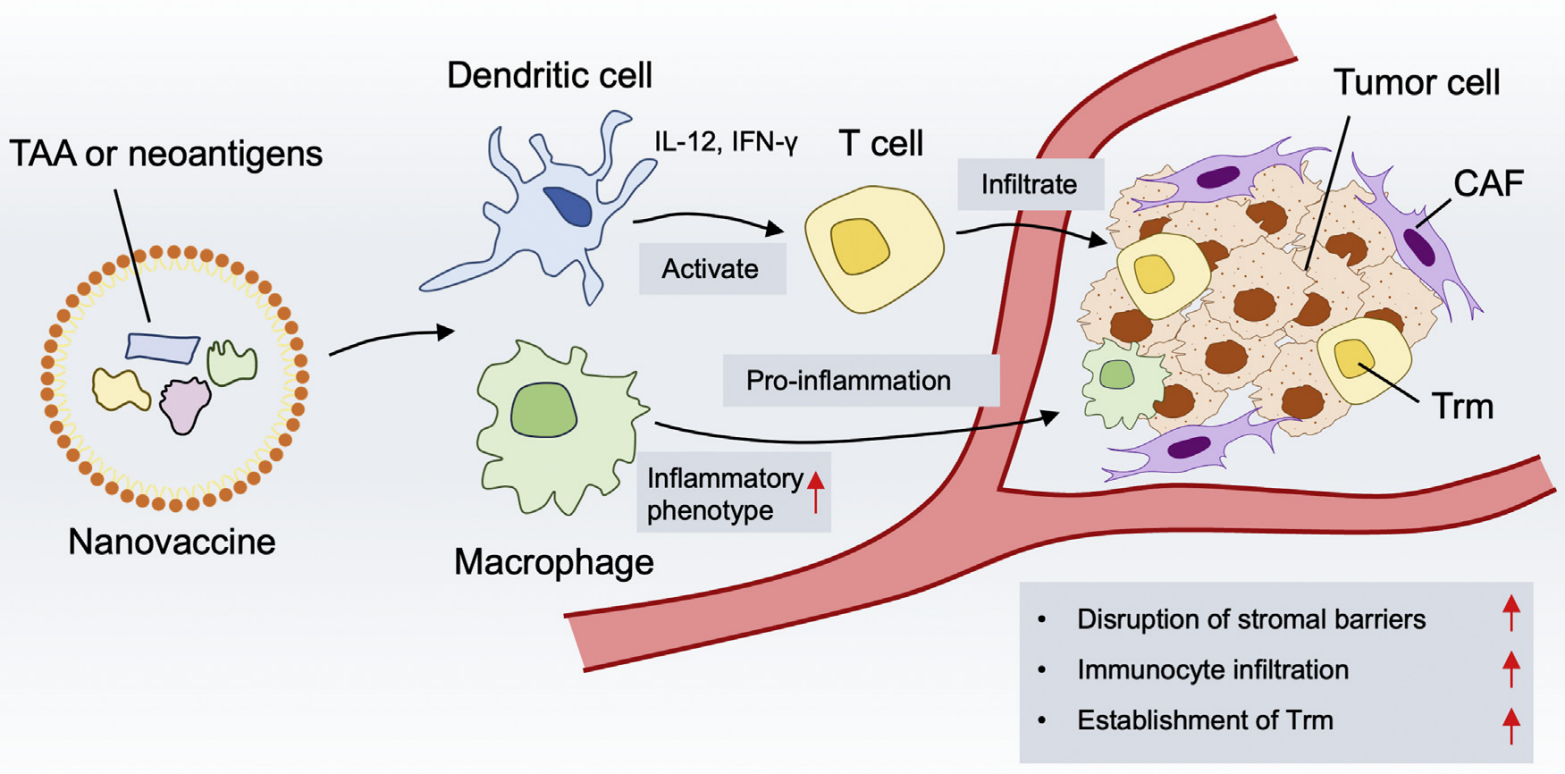
Immunological and biological basis of NVs. NVs carrying tumor-associated antigens (TAAs) or neoantigens could be taken up and then activate dendritic cells (DCs) and macrophages. Activated DCs secrete proinflammatory cytokines, stimulating T cell activation and infiltration into the tumor tissue. The internalized vaccines reprogram macrophages from immunosuppressive phenotype to proinflammatory phenotype. The combined proinflammatory effects of activated DCs and macrophages disrupt stromal barriers in the tumor microenvironment (TME), facilitating immune cell infiltration and enhancing establishment of tissue-resident memory T cells (Trm). CAF, Cancer-associated fibroblast; NVs, Nanovaccines.

Understanding the biological foundations of NVs is critical for optimizing their design and therapeutic efficacy in lung cancer.^22^ The efficacy of cancer NVs relies on presenting TAAs to immune system, overcoming the immunosuppressive TME, and triggering robust, long-lasting anti-tumor immune responses (Fig. 1).^23^ This section summarizes the key cellular and molecular behaviors underlying the mechanism of NVs in lung cancer immunotherapy, including TAAs, DCs, T cells, macrophages, and TME.

### Tumor antigens in lung cancer

Tumor antigens are central to the design and efficacy of NVs-based immunotherapies for lung cancer. These antigens are divided into TAAs and tumor-specific neoantigens, each presenting distinct opportunities that influence the magnitude and durability of anti-tumor immune responses.^22^^,^^23^

#### TAAs

TAAs are self-proteins that become aberrantly overexpressed, glycosylated, or dysregulated in cancer cells. In lung cancer, common TAAs include mucin-1 (MUC1), carcinoembryonic antigen (CEA), Survivin, Wilms’ tumor protein (WT1), and legumain.^24^, ^25^, ^26^ Due to their frequent and stable expression, these molecules are widely exploited in NV platforms. Packaging TAAs into nanocarriers including liposomes, polymeric NPs, or biomimetic vesicles improves DC uptake, facilitates lymph node delivery, and enhances antigen presentation. Co-delivery of TAAs with immune-stimulating adjuvants within the same NP further promotes DC activation and cross-presentation, leading to strong cytotoxic T lymphocyte (CTL) responses.^27^

However, TAAs are limited by their self-origin: central tolerance and peripheral immunoregulation could reduce TAA-specific T cell responses, while off-tumor toxicity remains potential risks. Strategies to circumvent these barriers include engineering more immunogenic TAA variants, designing multivalent formulations, and using adjuvants that break tolerance without triggering autoimmunity.^22^^,^^23^ In addition to NVs, some other nanomedicine strategies have been designed to polarize TAMs and thus reprogram the immunosuppressive TME.^28^

#### Neoantigens

Tumor-specific neoantigens are novel peptides generated by non-synonymous somatic mutations. Because they are absent from normal tissues, neoantigens escape central tolerance and exhibit high tumor specificity. Lung cancers, particularly those with smoking history or high tumor mutational burden (TMB), harbor a broad neoantigen repertoire that increases the probability of identifying immunogenic peptides.^29^^,^^30^ Advances in next-generation sequencing (NGS) and computational prediction have enabled the development of personalized NVs delivering patient-specific neoantigens such as synthetic peptides, mRNA, or tumor lysates. Early clinical studies in lung cancer have demonstrated that such vaccines can elicit potent, polyclonal T cell responses against diverse tumor subclones, thereby reducing immune escape due to antigen loss or intratumoral heterogeneity.^31^^,^^32^

Beyond personalized approaches, multi-antigen strategies employ whole-tumor lysate or tumor cell membrane as antigen sources, incorporating both shared TAAs and private neoantigens. These vaccines could induce both CD8⁺ and CD4⁺ T cells, establish lung tissue-resident memory T (Trm) cell responses, and provide cross-protection against heterogeneous metastatic lesions.^33^, ^34^, ^35^, ^36^ Cross-reactivity of antigens across tumor types, including melanoma and lung cancer, further supports the potential of broad-spectrum NVs.^37^

#### Innovative strategies

Some NVs incorporate cytotoxic drugs or small interfering RNA (siRNA) to induce ICD within tumors, thereby releasing additional neoantigens *in situ*.^38^ This approach triggers cyclic GMP-AMP synthase–stimulator of interferon genes (cGAS–STING) pathway and other immunomodulatory mechanisms to enhance the infiltration of immune cells into “cold” tumors.^39^ The physical characteristics of nanocarriers, particularly size, charge, and composition, critically shape intracellular antigen trafficking. NPs with sizes ranging from tens to a few hundred nanometers are most efficient for lymph node targeting and antigen cross-presentation.^40^

In the future, integration of multi-omics profiling, immunopeptidomics, and machine learning is expected to refine antigen prioritization and personalize NV design. Combining multi-antigen formulations and precise neoantigen targeting, while considering tumor evolution, is likely to yield the most durable immune protection in lung cancer.^41^, ^42^, ^43^

### Roles of DCs, T cells, and innate immunity

The therapeutic success of NVs in lung cancer extends beyond antigen formulation and depends on reshaping the function of immune cells that orchestrate anti-tumor responses. DCs, T cells, and innate immune effectors form the interconnected network, ultimately determining clinical efficacy.

#### DCs

Beyond their role as antigen carriers, DCs serve as critical sensors and regulators of vaccine-induced immunity. Their ability to migrate from peripheral tissues into draining lymph nodes has emerged as a strong predictor of clinical benefit in lung cancer immunotherapy.^44^ The abundance of migratory DCs, such as CD103⁺ DCs, which are indispensable for initiating effective anti-tumor T cell responses, is associated with improved outcomes in patients with immunotherapies.^44^ NV enhances DCs migration and function, therefore representing a promising avenue to achieve durable therapeutic responses.

#### T cells

Beyond effector cytotoxic responses, high-quality vaccines foster polyfunctional T cells to produce multiple cytokines and exhibit superior persistence. Establishing localized immune surveillance within the lung, mediated by Trm, is of equal importance. Trm populations are positioned within the pulmonary microenvironment to mount rapid responses against recurrent or metastatic lesions. The presence of Trm after vaccination has been linked to long-term tumor control in preclinical and clinical models.^45^

#### Innate immunity

The broader innate immune system also sets the stage for adaptive immune success. Engagement of pattern recognition receptors by NVs-associated molecular motifs induces strong type I interferon responses and chemokine secretion, particularly Toll-like receptors (TLRs) and the cGAS–STING pathway.^46^^,^^47^ Innate effector cells such as macrophages, natural killer (NK) cells and neutrophils contribute to the early containment of tumor growth and shape the subsequent adaptive response.^48^^,^^49^ Therefore, incorporating innate agonists into vaccine platforms has become rational strategies to broaden and intensify anti-tumor immunity. TAMs in lung cancer display immunosuppressive phenotypes, releasing interleukin 10 (IL-10) and transforming growth factor-β (TGF-β) and fostering angiogenesis and extracellular matrix remodeling, correlating with poor prognosis and resistance.^50^ Next-generation NVs are designed to deplete immunosuppressive TAMs or reprogram them to proinflammatory phenotypes. NPs with ligands or agents modulating macrophage polarization, convert the tumor milieu from suppressive to immunostimulatory. By reversing TAMs-driven suppression, these vaccines restore T cell infiltration and amplify the cytotoxic cascade within tumors.^50^^,^^51^

#### Integrated orchestration

The significance of NVs lies not in activating single immune cell types alone but in synchronizing their actions to remodel the TME. Synergistic modulation of DCs, T cells, and innate effectors has the potential to transform immunologically “cold” lung cancers into “hot” inflamed lesions receptive to long-term immune surveillance.^52^ Importantly, this reprogramming effect also enhances response to immune checkpoint inhibitors, highlighting the potential of NVs as a cornerstone in combination immunotherapy strategies.

### Tumor microenvironment and immune evasion mechanisms

The TME in lung cancer represents a multifaceted barrier that extends well beyond the malignant cells themselves, which comprises extracellular matrix (ECM) components, cancer-associated fibroblasts (CAFs), endothelial cells, soluble mediators, and metabolic byproducts that collectively shape the immune landscape. This system not only facilitates tumor progression and metastatic dissemination, but also impairs the capacity of immune interventions to achieve durable control.^53^

A defining feature of the TME is physical and structural complexity. CAFs and the deposition of dense ECM proteins, such as collagen and fibronectin, generate a stiff and fibrotic stroma that restricts the penetration of immune cells and therapeutic agents into the tumor core.^54^ This architectural barrier is further reinforced by aberrant vasculature, which leads to heterogeneous blood flow, hypoxic niches, and uneven nutrient distribution. Hypoxia itself drives the stabilization of hypoxia-inducible factor 1-alpha (HIF-1α), promoting angiogenesis and metabolic adaptation, and upregulating immunosuppressive mediators.^55^^,^^56^

At the molecular level, lung cancer cells exploit multiple immune evasion strategies that directly impair recognition by cytotoxic lymphocytes. This process includes the upregulation of inhibitory checkpoint ligands such as programmed cell death ligand 1 (PD-L1), T-cell immunoglobulin and mucin-domain containing-3 (TIM-3), and lymphocyte-activation gene 3 (LAG-3), which induce T cell exhaustion, as well as the downregulation or complete loss of major histocompatibility complex (MHC) molecules, which limit antigen visibility.^17^^,^^57^ In parallel, tumor-derived metabolites, particularly adenosine and lactic acid, reshape the biochemical milieu, blunting effector cell function and favoring tolerance.^58^

The adaptive resistance of the TME further complicates therapeutic efforts. Under selective pressure from immunotherapies, tumor subclones undergo immunoediting, leading to antigen loss variants or the emergence of resistant lineages. Moreover, conventional treatments such as chemotherapy and radiotherapy alter the stromal architecture, sometimes intensifying immunosuppressive features or triggering compensatory inhibitory pathways.^53^^,^^59^

Innovative NV strategies are being developed to address these unique challenges. Platforms incorporating biomimetic coatings, including those derived from tumor cell membranes, bacterial membranes, or hybrid constructs, enable NPs to preferentially accumulate within the tumor niche while simultaneously delivering endogenous or exogenous danger signals.^60^^,^^61^ Other approaches combine vaccine payloads with cytokines or chemokines that disrupt stromal barriers, normalize vasculature, or promote immune infiltration into inaccessible tumor regions.

In addition to their role in delivery, NVs undergo a series of dynamic interactions with immune system cells and the TME after administration. Once processed by antigen presenting cells (APCs) such as DCs, NVs facilitate antigen processing and cross-presentation, ensuring T cell priming and expansion. Immune activation induced by NVs can also modify the TME by reducing immune-suppressive signals, promoting a pro-inflammatory status in TAM, and increasing infiltration by CTLs.^62^, ^63^, ^64^ In hours to days, NVs support a highly efficient antigen drainage to lymph nodes or direct uptake by APCs. This leads to rapid DC maturation and migration, indicated by an increased expression of costimulatory molecules such as CD80/86 and the production of pro-inflammatory cytokines such as interleukin 12 (IL-12) and interferon γ (IFN-γ). Naive T cells are thereby primed, and antigen-specific expansion of CD8⁺ CTL is initiated.^23^^,^^65^ In the intermediate phase, approximately 3–14 days after treatment, these activated CTL and CD4⁺ T helper lymphocytes migrate into the TME where NV treatment leads to a massive TME remodeling. Most advanced NV systems include immune-modulatory drugs such as STING agonists or colony-stimulating factor 1 receptor (CSF1R) inhibitors targeting TAM, thus enhancing pro-inflammatory pathways while downregulating the production of anti-inflammatory cytokines.^66^^,^^67^ In addition, depletion or pharmacologic inhibition of regulatory T cells (Tregs) and myeloid-derived suppressor cells (MDSCs) suppresses immune exhaustion, which leads to an increased influx of CTL into pre-established “cold tumors”.^68^ Weeks to months later, a sustained immune memory is established. Tissue-resident memory T-cells accumulate in lungs, mounting a quick recall response against secondary metastases.^69^

Despite these advances, the heterogeneity and plasticity of the TME mean that immune evasion remains a moving target. Lung cancer progression is promoted not only by the presence of immunosuppressive factors but also by their adaptive resistance to therapy.^59^ As such, the next generation of lung cancer NVs must integrate context-specific strategies that account for stromal barriers, metabolic constraints, and immune escape pathways. This systems-level approach is essential to transform the TME from an obstacle into an ally of durable antitumor immunity.

## NV platforms in lung cancer

**Fig. 2:**
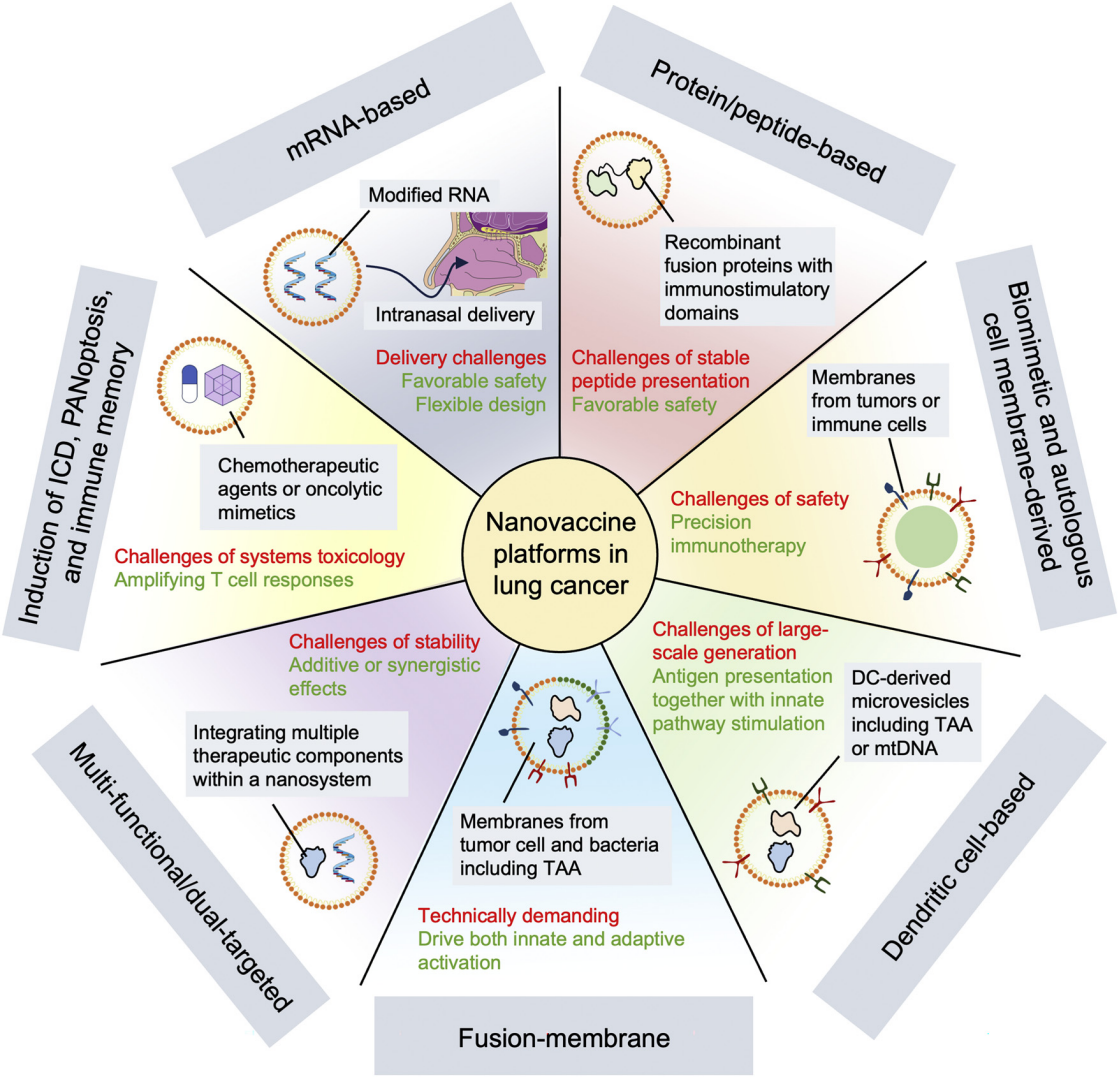
NV platforms in lung cancer. (1) mRNA-based vaccines: contain modified RNA and could be administered via intranasal delivery, offering flexible design and favorable safety; (2) protein/peptide-based vaccines: employ recombinant fusion proteins with immunostimulatory domains, exhibiting safety but limited stability; (3) biomimetic and autologous cell membrane-derived vaccines: utilize membranes from tumor or immune cells, driving precise medicine but safety concerns; (4) dendritic cell-based vaccines: rely on DC-derived microvesicles, activating both innate and adaptive immunity; (5) fusion-membrane vaccines: combine tumor and bacterial membranes, driving robust innate and adaptive immune activation; (6) multi-functional/dual-targeted vaccines: integrate photosensitizers or siRNA to achieve synergistic immune and cytotoxic effects; (7) ICD/PANoptosis-based vaccines: induce immunogenic cell death or PANoptosis via chemotherapeutic or photothermal agents. Labels in red indicate challenges or disadvantages, whereas labels in green indicate advantages. DC, Dendritic cell; ICD, Immunogenic cell death; mRNA, Messenger RNA; mtDNA, Mitochondrial DNA; NV, Nanovaccine; PANoptosis, Pyroptosis, apoptosis, and necroptosis; siRNA, Small interfering RNA; TAA, Tumor-associated antigen.

Overcoming the formidable barriers imposed by the TME and diverse immune evasion pathways remains a central challenge in lung cancer therapy.^70^ While conventional immunotherapeutic approaches have achieved only limited success against the adaptive and suppressive features, the emergence of NV technologies has introduced new opportunities for more precise and effective modulation of anti-tumor immunity (Fig. 2).^22^ By harnessing the unique properties of nanomaterials and advanced delivery systems, current NVs are designed to not only deliver tumor antigens efficiently but also to reshape the immune landscape within the tumor, enhance antigen presentation, and reverse the local immunosuppression.^71^

### mRNA-based NVs

Messenger RNA (mRNA)-based NVs have emerged as a transformative platform in cancer immunotherapy due to their versatility, rapid manufacturability, and capacity for personalization.^72^^,^^73^ Unlike conventional peptide or protein vaccines, mRNA formulations provide a blueprint that can be readily adapted to encode multiple targets, enabling flexible vaccine design in response to the evolving landscape of lung cancer. The modularity of the technology allows rapid incorporation of new antigenic sequences, streamlined synthesis, and scalable production processes that are well-suited for both individualized and off-the-shelf applications.^74^

The success of mRNA vaccination relies heavily on advances in delivery systems that stabilize otherwise fragile RNA molecules. Lipid NPs (LNPs) remain the most widely used carriers, engineered to shield mRNA from degradation, promote endosomal escape, and ensure efficient translation in host cells.^75^, ^76^, ^77^ Mai et al^78^ investigated the use of synthetic mRNA encoding cytokeratin 19 (CK19), a common TAA in lung cancer, delivered intranasally using a cationic liposome-protamine complex (LPC) to address current issues such as mRNA instability, insufficient cellular uptake, and inadequate immune responses. Beyond classical LNPs, ongoing research explores polymeric carriers, hybrid NPs, and bioinspired designs optimized for biodistribution, pharmacokinetics, and selective uptake by immune-relevant tissues.^79^ A novel circular RNA (circRNA) vaccine encapsulated in lipid NPs was tested in both prophylactic and therapeutic settings against lung metastasis. The intranasal prime-boost regimen, administered at weekly intervals, demonstrated potent immunogenicity. In B16-ovalbumin (OVA) and LL/2 pulmonary metastasis models, prophylactic vaccination reduced metastatic foci by >60% relative to mock controls and significantly lowered composite lung tumor indices.^80^ In therapeutic experiments, mice receiving circRNA-LNP after tumor challenge exhibited a pronounced decrease in metastatic nodules and an extended survival advantage of ∼20 days compared to untreated animals. Immunological assays revealed strong expansion of OVA-specific CD8⁺ T cells, elevated IFN-γ and granzyme B secretion, and enrichment of CD8⁺ central memory populations. Importantly, the intranasal route facilitated localized mucosal immunity, evidenced by elevated immunoglobulin A (IgA) titers in bronchoalveolar lavage fluid. No systemic toxicity or weight loss was observed, highlighting the safety of repeated dosing. Collectively, these data illustrate how circRNA NVs establish both systemic and mucosal memory responses, offering a versatile tool for NSCLC therapy.^80^ BNT116 is an encapsulated mRNA vaccine developed by BioNTech and encodes NSCLC relevant TAAs, which is designed for both monotherapy and combination with immune checkpoint inhibitors.^81^ The first-in-human Phase I trial (NCT05142189) is recruiting advanced/metastatic NSCLC patients to assess dose escalation, tolerability, and immunogenicity. Early safety reports reveal that treatment-related adverse events are mostly grade 1–2, with flu-like symptoms, mild injection-site reactions, and most common transient fatigue. Importantly, no dose-limiting toxicities (DLTs) or grade ≥ 3 vaccine-related immune adverse events have been documented to date, demonstrating a favorable safety profile consistent with other mRNA-based NVs. Though ongoing, preliminary immunogenicity assessments have shown expansion of antigen-specific CD8⁺ T cells and polyfunctional T cell subsets capable of secreting IFN-γ, tumor necrosis factor α (TNF-α), and interleukin 2 (IL-2).^81^ The Phase II EMPOWERVAX Lung 1 trial (NCT05557591) is testing BNT116 in combination with cemiplimab as first-line therapy for PD-L1 ≥ 50% NSCLC patients. The trial design randomizes participants to cemiplimab alone versus cemiplimab plus BNT116, with primary endpoint of overall response rate (ORR) and key secondary endpoints including progression-free survival (PFS). Choice of high PD-L1 cohort mirrors checkpoint blockade responsiveness, allowing evaluation of added benefit from mRNA nanovaccination. Although efficacy data remain pending, translational endpoints such as induction of circulating tumor-specific T cells, changes in tumor infiltrating lymphocyte density, and modulation of the TME are being collected, positioning BNT116 as a highly advanced NV candidate in NSCLC.^82^

V940 (mRNA-4157) represents one of the highly ambitious efforts to bring personalized mRNA vaccines into the NSCLC adjuvant setting. Encapsulated in LNPs, the vaccine encodes up to 34 neoantigens per patient, which are identified by exome sequencing and bioinformatic prediction pipelines. The Phase III INTerpath-002 trial (NCT06077760) randomizes patients with resected stage II–III NSCLC to pembrolizumab alone versus pembrolizumab plus V940.^83^ Key endpoints include disease-free survival (DFS), overall survival (OS), and immune correlates, such as neoantigen-specific T cell expansion, cytokine profiling, and T cell receptor (TCR) clonotype diversity. The rationale stems from earlier melanoma trials (KEYNOTE-942), where the same vaccine platform reduced recurrence risk by 44% compared to pembrolizumab alone, with durable immune responses lasting beyond 18 months.^84^ The Phase III trial represents a decisive test for integrating personalized mRNA NVs into curative-intent lung cancer care.^85^ The mRNA-5671 (V941) vaccine is developed by Moderna and as a model of mutation-specific precise NVs. Delivered via LNPs, the vaccine encodes peptides corresponding to the four most common *KRAS* mutations in NSCLC (G12C, G12D, G12V, G13D). The Phase I trial (NCT03948763) enrolled patients with advanced NSCLC harboring *KRAS* mutations, testing both monotherapy and combination with pembrolizumab.^86^ Preliminary results demonstrate excellent tolerability, with adverse events limited to mild injection-site erythema, transient fever, and fatigue, all grade 1–2 in severity. Immunological analyses revealed expansion of *KRAS* mutation-specific CD8⁺ T cells in peripheral blood within 2–4 weeks post-vaccination. In a subset of patients, vaccine-induced T cells exhibited polyfunctional profiles, producing IFN-γ, granzyme B, and TNF-α, while maintaining memory-associated markers such as CD127. Importantly, early signals of clinical benefit have emerged, including partial responses (PRs) in a subset of pretreated patients and durable stable disease (SD) in others. This trial is one of the first to clinically demonstrate that mRNA NV can selectively expand T cells against oncogenic driver mutations, paving the way for combinatorial approaches with *KRA*S G12C inhibitors or checkpoint blockade in NSCLC.

A distinctive area of innovation is intranasal delivery, which has particular relevance for lung malignancies. By engaging the mucosal immune system of the respiratory tract, intranasal formulations can establish localized immunity directly at the site of disease initiation and progression.^87^ Specialized nanoformulations, including cationic lipids, mucoadhesive polymers, or inorganic carriers, facilitate adherence to airway surfaces and penetration across epithelial barriers, enhancing vaccine retention and bioactivity in pulmonary tissues.^88^ Li et al^87^ designed a layered double hydroxide (LDH) NP system for intranasal delivery of E7-encoding mRNA, combined with the mucosal-associated invariant T (MAIT) cell agonist 5-OP-RU. This dual-activation design aimed to remodel the lung immune microenvironment by engaging both conventional DCs and innate-like T cells. In TC-1 pulmonary metastasis models, three intranasal doses (administered on days 3, 6, and 10 post-tumor inoculation) elicited a significant increase in proinflammatory cytokines and chemokines, such as IFN-α, IFN-γ, and C-X-C motif chemokine ligand 10 (CXCL10), as well as a significant decrease in anti-inflammatory cytokines, including TGF-β and IL-10. These cytokine or cytokine shifts were paralleled by increased infiltration of CD8⁺ T cells and a higher inflammatory/anti-inflammatory macrophage ratio in lung tissue, shifting the TME from a suppressive to a more stimulatory milieu. Tumor burden analysis confirmed significantly fewer visible lung nodules in treated mice versus naked mRNA controls. Additionally, flow cytometry revealed expansion of effector and central memory T cells, supporting durable systemic protection. These results underscore the synergy between mRNA delivery and MAIT-cell engagement, positioning LDH-based intranasal NVs as a next-generation modality for both therapeutic and prophylactic intervention in lung cancer.^87^

Chemical refinements of mRNA molecules further strengthen this platform. Incorporation of modified nucleosides improves translation efficiency while mitigating unwanted activation of innate sensors that otherwise limit expression of the proteins encoded by the mRNAs.^89^ Coupled with optimized capping strategies and codon usage, these refinements extend the half-life of mRNA *in vivo* and support robust protein synthesis.^90^

From a clinical perspective, mRNA NVs demonstrate favorable safety profiles (Table 1). They avoid the risks of genomic integration associated with DNA-based platforms, and their transient expression reduces concerns of long-term persistence.^74^ Moreover, the ability to integrate multiple functional modules provides opportunities to fine-tune both efficacy and safety, including co-delivered immune agonists, cytokine-encoding transcripts, or targeting ligands.^79^ However, several challenges remain, including mRNA instability (partially mitigated by LNPs), potential innate immune overactivation, and the higher costs for personalized formulations.

**Table 1:** mRNA- or peptide-based vaccines in clinical trials for NSCLC.

Vaccine	Type	NCT	Phase	Lung cancer type	Intervention	Patients (*n*)	Primary endpoints	Secondary endpoints	Recruitment status	Expected/actual completion
Personalized mRNA neoantigen vaccine	mRNA-based	NCT03908671	Early/ pilot	Advanced NSCLC (also esophageal cancer)	Personalized mRNA vaccine encoding patient-specific neoantigens	24	Safety, tolerability	Preliminary efficacy, ORR, DCR, PFS, OS	Recruiting	Dec 2025
CVHNLC	mRNA-based	NCT07073183	Early (Phase 1)	NSCLC	CVHNLC mRNA vaccine combined with pembrolizumab	36	Treatment-related adverse events, DLTs, safety	ORR, PFS, DoR, disease control rate	Not yet recruiting	Dec 2029
BMD006	mRNA-based	NCT06928922	Phase 1	Advanced lung cancer/lung metastases	Inhaled dry-powder mRNA vaccine ± PD-1 antibody	22	DLTs, MTD/RP2D, safety	ORR, DCR, PFS, OS, immune responses, ctDNA dynamics	Recruiting	Feb 2028
BI 1361849 (CV9202)	mRNA-based	NCT03164772	Phase 1/2	Metastatic/advanced NSCLC	BI 1361849 + durvalumab ± tremelimumab	61	Safety, treatment-emergent adverse events	PFS, tumor response, DoR	Completed	Oct 2021
mRNA-5671 (V941)	mRNA-based	NCT03948763	Phase 1	*KRAS*-mutant NSCLC (and other solid tumors)	*KRAS*-targeted mRNA vaccine ± pembrolizumab	70	DLTs, safety, treatment discontinuation due to AEs	ORR, KRAS-specific T-cell responses	Terminated	Aug 2022
RGL-270	mRNA-based	NCT06685653	Phase 2	NSCLC (resectable and advanced)	Personalized neoantigen mRNA vaccine + adebrelimab	65	DLTs, safety	Neoantigen-specific T-cell responses, ORR, DFS, OS, PFS	Not yet recruiting	Nov 2026
Personalized mRNA neoantigen vaccine	mRNA-based	NCT06735508	Early Phase 1	Resected NSCLC	Personalized mRNA neoantigen vaccine + adebrelimab (adjuvant)	40	Safety, DLTs, MTD/MAD	Immunogenicity, DFS, OS	Not yet recruiting	Dec 2026
LuCa-MERIT-1 (BNT116)	mRNA-based	NCT05142189	Phase 1	Advanced/resectable NSCLC	BNT116 alone or combined with cemiplimab, chemotherapy, or antibody conjugates	280	DLTs, safety, tolerability	ORR, DoR, PFS, OS, EFS, pathological response rates	Recruiting	Nov 2031
Tecemotide (L-BLP25)	Protein/peptide-based	NCT00409188	Phase 3	Unresectable stage III NSCLC after CRT	Tecemotide + cyclophosphamide *vs*. placebo	1513	OS	TTP, symptom progression, PROs, safety	Completed	Apr 2015

AEs, Adverse events; CRT, Chemoradiotherapy; ctDNA, Circulating tumor DNA; DCR, Disease control rate; DFS, Disease-free survival; DLTs, Dose-limiting toxicities; DoR, Duration of response; EFS, Event-free survival; MAD, Maximum administered dose; mRNA, Messenger RNA; MTD, Maximum tolerated dose; NSCLC, Non-small cell lung cancer; ORR, Objective response rate; OS, Overall survival; PD-1, Programmed cell death protein 1; PFS, Progression-free survival; PROs, Patient-reported outcomes; RP2D, Recommended phase 2 dose; TTP, Time to progression.

Future development is increasingly focused on overcoming delivery challenges specific to lung cancer, including efficient deposition in respiratory tissues and navigation of physiological barriers. Strategies that integrate mRNA vaccination with complementary therapies, leverage real-time genomic profiling, and exploit nanotechnology-driven design principles are expected to establish this modality as a central component of next-generation lung cancer immunotherapy.

### Protein/peptide-based NVs

Protein and peptide-based NVs constitute a versatile platform that emphasizes precise engineering of antigen formats and carrier systems.^91^ In lung cancer, these vaccines are valued for incorporating well-defined molecular components into nanoscale delivery vehicles, allowing safety and reproducibility, which are distinct from nucleic acid-based or live-vector approaches.^92^ The antitumor activity of peptide-based NVs is illustrated in a B16-OVA lung metastasis model. Here, these models received six intranasal doses of a polymer-antigen-adjuvant nanocomplex (DA3/R848/OVA) on days 2, 4, 6, 8, 10, and 12 following intravenous tumor inoculation. This treatment significantly reduced the number of metastatic foci, total lung weight, and a composite tumor index compared with untreated or OVA-only controls. Notably, inclusion of the TLR7/8 agonist (R848) and nanocarrier was essential, as OVA alone had no therapeutic effect. Vaccination also extended survival by ∼7 days compared with controls and increased splenic CD8⁺ central memory cells, demonstrating durable memory formation.^93^

An inhalable NV was engineered using dextran-derived polysaccharide NPs (DDP) to encapsulate lung tumor antigens. *In vitro* assays demonstrated that DDP promoted robust DCs maturation, as evidenced by elevated CD80/CD86 expression and secretion of IL-12 and TNF-α. *In vivo*, prophylactic vaccination in a Lewis lung carcinoma (LLC) model yielded striking results: 50% of vaccinated mice survived beyond 180 days, whereas all controls succumbed before day 90. Notably, rechallenge experiments revealed that 33% of survivors remained tumor-free after secondary LLC inoculation, providing functional evidence of durable immune memory. Compared with subcutaneous delivery, the intranasal route significantly enhanced systemic CD4⁺ and CD8⁺ T cell expansion, increased immunoglobulin G (IgG) titers against tumor antigens, and enriched lung-resident memory T cells. Importantly, histopathological analyses confirmed the absence of lung toxicity, underlining the safety of repeated pulmonary dosing. Together, these findings establish inhaled polysaccharide-based NVs as a clinically relevant platform capable of inducing long-lasting, protective immunity specifically suited for lung cancer.^94^

Liposomes are the most promising lipid nanoplatforms and widely used in the treatment of lung cancer. There are two key aspects to consider when developing liposomes for vaccine delivery: the encapsulation of vaccine and the modification of their surfaces. LPC showed significantly improved efficacy in the uptake of vaccine particles *in vitro* and demonstrated superior abilities to promote the maturation of DCs, which subsequently triggered a robust anti-tumor immune response. In a highly aggressive LLC mouse model, intranasal administration of cationic LPC with mRNA encoding CK19 showed a marked inhibition of tumor growth. Analysis of the spleen showed an increase in CD4^+^ and CD8^+^ T cells and IL-4 and IL-2, confirming a strong systemic T cell activation.^78^ The findings of this investigation offer compelling evidence that cationic LPC serves as a safe and effective adjuvant, and this mRNA formulation lays the groundwork for anti-cancer vaccination in humans. Research showed that the BLP25 vaccine (L-BLP25, Stimuvax®), which specifically targets MUC1/CD227, an antigen overexpressed and misglycosylated in lung cancer, has potential for the treatment of NSCLC.^95^^,^^96^ A randomized Phase IIB clinical trial evaluated the efficacy of L-BLP25 in combination with palliative treatment versus palliative treatment alone in 171 patients with stage IIIB/IV NSCLC, who showed no evidence of disease progression after initial chemotherapy or chemoradiotherapy. Preliminary results showed that the median survival time for patients who received L-BLP25 together with palliative care was 17.4 months, compared to 13.0 months for patients who received palliative care alone. However, the difference between the treatment groups could not achieve statistical significance. In addition, L-BLP25 was well tolerated by patients with stage IIIB/IV NSCLC, with most adverse events associated with the disease rather than the investigational product.^97^^,^^98^ The most adverse events were mild flu-like symptoms, and only one case showed pneumonia related to vaccine.^97^ However, certain results are not encouraging, as shown in another clinical trial comparing L-BLP25 with placebo or cyclophosphamide in patients with unresectable stage III NSCLC (NCT00409188), which found no significant difference in OS.^99^ Potential mechanisms underlying this failure in the broader population include the self-origin of MUC1 as a TAA, leading to central thymic tolerance and peripheral tolerance and suboptimal breaking of immune ignorance without sufficient adjuvant potency or optimal timing relative to chemoradiotherapy-induced immunogenic signals. Additionally, heterogeneity prior to therapy exposure, tumor mutation burden, and immune evasion mechanisms (e.g., Treg infiltration, PD-L1 expression) may also have diluted efficacy.^100^ Furthermore, the Phase IB trial of NEO-PV-01, a personalized peptide vaccine, integrated computational neoantigen prediction with clinical immunology in NSCLC patients. Synthetic long peptides were formulated with the polyinosinic–polycytidylic acid stabilized with poly-L-lysine and carboxymethylcellulose (poly-ICLC) adjuvant and co-administered alongside chemotherapy and pembrolizumab. Out of the 82 patients enrolled in the study, which encompassed various solid tumors, 12 patients were diagnosed with advanced NSCLC. In this subgroup, vaccine-induced neoantigen-specific T cell responses were observed in 83% of patients, with both CD4⁺ helper and CD8⁺ cytotoxic subsets activated. Importantly, many vaccine-elicited T cell clones persisted up to 96 weeks after vaccination, indicating long-lasting immune memory. Functional assays demonstrated that 75% of patients developed polyfunctional CD8⁺ T cells producing multiple cytokines simultaneously. Single-cell RNA sequencing confirmed expansion of novel TCR clonotypes not detectable pre-vaccination. Clinically, while the study was not powered for efficacy, several NSCLC patients experienced prolonged progression-free intervals relative to historical benchmarks, with some patients maintaining disease control beyond 12 months. Furthermore, the safety of vaccination was excellent, without grade ≥3 toxicities observed. Incomplete neoantigen coverage, and suboptimal induction of CTL may be key reasons for ambiguity regarding efficacy. These results provide clinical proof-of-concept that personalized peptide NVs overcome tumor heterogeneity in NSCLC by broadening the repertoire of tumor-specific immune responses.^101^

Recombinant protein NVs add a further dimension of precision. Advances in molecular biology enable the generation of purified proteins or synthetic constructs engineered to display optimized epitopes, trafficking motifs, or immune-enhancing sequences. These constructs can be organized into self-assembling NPs, micelles, or scaffolds, creating defined architectures with consistent antigen density and orientation.^102^ Such control facilitates reliable vaccine performance and the possibility of integrating multiple design features within a single system, such as adjuvant domains or multivalent peptide arrays.^92^ Emmers et al^103^ developed T-cell epitopes associated with impaired peptide processing 24 (TEIPP24), a synthetic long peptide vaccine derived from low-density lipoprotein receptor-related protein associated protein 1 (LRPAP1), which is intended to boost T cell immunity in NSCLC. They investigated the safety, tolerability, and immunomodulatory effect of the vaccine as well as the antigen and immune profiles of patients in a clinical trial of human leukocyte antigen (HLA)-A*0201-positive patients with NSCLC following treatment with pembrolizumab (NCT05898763). Treatment was well received by all 26 participants, with a vaccine-induced LRPAP1_21-30_-specific CD8^+^ T cell response observed in over 80% of cases, and 62% of patients also had vaccine-specific CD4^+^ T cells. The increase of activated polyfunctional CD8^+^ effector T cells was influenced by vaccine dose, induction of CD4^+^ T cell response, and baseline monocyte frequency. In an *ex vivo* setting, co-administration of pembrolizumab led to the identification of activated (human leukocyte antigen-DR^+^ [HLA-DR^+^], programmed cell death protein 1^+^ [PD-1^+^], inducible T-cell co-stimulator^+^ [ICOS^+^]) LRPAP1-specific CD8^+^ T cells and thus created a solid basis for the further development of the TEIPP targeting concept in tumor therapy. The observation of one PR, eight stable diseases (SDs), and two mixed responses in 24 evaluable patients after vaccination was associated with a robust CD8^+^ T cell response to this singular epitope from this novel category of cancer antigens.^103^ Wang et al^104^ developed a *KRA*S-targeted vaccine containing peptides from both mutant and wild-type *KRAS*, combined with a nanoemulsion adjuvant (NE). This formulation was tested in a mouse model with inducible mutant *KRAS*-driven lung cancers. Mice vaccinated intranasally with the NE-adjuvanted KRAS peptides showed a decrease in the populations of CD4^+^forkhead box protein P3 (FoxP3)^+^ T cells in their lymph nodes and spleen. In addition, these animals showed increased secretion of IFN-γ and IL-17a in response to the *KRAS* mutation, mediated primarily by CD8^+^ T cells, as well as sustained *KRAS*-specific Th1 and Th17 immune activation that persisted for 3 months after the last dose. Remarkably, the vaccinated mice showed a significant reduction in tumor development rates compared to controls.^104^

An advantage of protein and peptide-based NVs lies in their favorable safety profile. By avoiding replicating vectors or genetic material, they minimize risks of genomic alteration while providing predictable pharmacokinetics.^91^ Their modularity also supports rapid adaptation to new molecular targets and facilitates combination with complementary nanotechnologies. Nevertheless, key challenges remain, particularly in achieving stable peptide presentation, fine-tuning release kinetics, and ensuring efficient lymphatic trafficking. Moreover, their effectiveness is often limited by relatively low intrinsic immunogenicity, requiring strong adjuvants, as well as HLA restrictions and challenges in addressing tumor heterogeneity resulting from predetermined epitopes. In contrast, mRNA-based NVs can induce strong, long-lasting, and polyfunctional T cell memory responses, with reduced HLA dependence and greater flexibility in addressing tumor heterogeneity by encoding full-length antigens or multiple neoantigens.^105^^,^^106^ To address the limitations of peptide-based NVs, recent efforts have explored multi-epitope formulations, incorporation of helper motifs, and designing smart release systems capable of responding to local physiological cues.^102^

In summary, protein and peptide-based NVs are emerging as adaptable components of the broader immunotherapy landscape. Their emphasis on structural engineering, modularity, and safety positions them as promising candidates for integration into next-generation nanomedicine platforms for lung cancer.

### Biomimetic and autologous cell membrane-derived NVs

Biomimetic and autologous cell membrane-derived NVs represent one of the most advanced directions of cancer vaccines, aiming to replicate the structural and immunological complexity of natural cells within nanoscale systems.^107^ Unlike synthetic or recombinant antigen-based approaches, these vaccines exploit entire cell membranes, sourced from tumors, immune cells, or other biological systems, to endow NPs with molecular fingerprints that are closer to the *in vivo* antigenic reality of cancer. This strategy introduces a level of authenticity and diversity that conventional platforms, limited by predefined antigens or epitopes, cannot easily achieve.^36^

A central innovation of this approach lies in the use of tumor cell membranes to cloak NPs. By transferring the full molecular repertoire of a tumor cell onto the NP surface, including proteins, lipids, carbohydrate modifications, and even patient-specific mutational signatures, these constructs act as “nano-tumor mimics”. This broad-spectrum antigenic display increases the chances of generating polyclonal immune recognition across heterogeneous tumor populations, including rare or mutationally evolved clones. Importantly, because antigens are presented in their natural conformation and orientation within the lipid bilayer, the resulting immune response is thought to more accurately reflect the biology of the tumor.^108^ Fusciello et al^109^ developed an oncolytic adenovirus cloaked with homologous tumor membranes (ExtraCRAd) and tested it across lung cancer xenograft and LLC models. Intratumoral delivery of 1 × 10^9^ viral particles enhanced oncolysis, increased intratumoral CD4⁺ and CD8⁺ T cell infiltration, and expanded APC and CD8⁺ T cell populations in draining lymph nodes. As a preventive vaccine, ExtraCRAd extended survival with >50% of mice alive at day 40, while significantly delaying tumor growth compared to naked virus or heterologous membrane controls, demonstrating the value of tumor-mimicking NVs in generating durable antitumor immunity. Miao et al^110^ introduced an inhalable biomimetic NV (BMVax), which was constructed from *E. coli*-derived bacterial membrane vesicles (BMVs) engineered to express the model tumor antigen OVA (ClyA-OVA). This strategy was designed to exploit the pulmonary mucosal route for direct immune priming in lungs. In a B16-OVA pulmonary metastasis model, intranasal administration of BMVax achieved complete prevention of lung metastases in 83.3% of treated mice, while all untreated controls developed numerous metastatic nodules. Quantitative immune profiling revealed that inhaled delivery was superior to subcutaneous administration: germinal center B cells in bronchial draining lymph nodes increased by 5.8-fold, follicular helper T cells by 4.9-fold, and mature DCs by 2.5-fold. Moreover, the frequency of OVA-specific CD8⁺ T cells in the lung was nearly three times higher following inhaled vaccination, accompanied by a marked rise in IFN-γ secretion and cytotoxic function. Flow cytometry confirmed expansion of memory T cell subsets, while bronchoalveolar lavage fluid showed increased IgA titers, highlighting the induction of both systemic and mucosal immunity. Importantly, vaccinated mice remained protected upon tumor rechallenge, demonstrating durable immune memory. These quantitative findings establish that bacterial membrane-derived NVs delivered via the respiratory tract robustly activate humoral and cellular immunity, providing a highly effective strategy for the prevention and treatment of lung metastasis.

Beyond tumor-derived materials, immune cell membranes have also been harnessed to enhance functional capabilities. NPs coated with DC membranes leverage natural antigen-presentation and immune-activating signals, while those derived from macrophage membranes may provide enhanced targeting to inflammatory or tumor-associated tissues. Similarly, red blood cell membranes have been explored to extend systemic circulation and evade rapid clearance, making them attractive scaffolds for sustained delivery of vaccine.^111^ Fu et al^112^ recently developed Bio-HCP@FM-NPs, a bifunctional biomimetic NV designed to synergize pathogen-associated molecular patterns with TAAs for lung cancer. The core consisted of hyper-crosslinked polymeric NPs encapsulating granulocyte-macrophage colony stimulating factor (GM-CSF), cloaked with a hybrid fusion membrane combining bacterial inner-membrane fragments from *E. coli* and senescent tumor cell membranes. This dual-membrane strategy provided both adjuvanticity and broad-spectrum tumor antigen presentation. *In vitro*, Bio-HCP@FM-NPs strongly promoted DC activation: surface expression of CD80 and CD86 was increased by ∼1.8-fold compared with control NPs, and cytokine assays revealed a 2.1-fold increase in IL-12 and a 1.6-fold increase in TNF-α secretion. Enhanced uptake of TAAs by DCs was also documented, resulting in superior cross-presentation efficiency. *In vivo*, administration in LLC models significantly delayed tumor progression. When combined with anti-PD-1 checkpoint blockade, tumor growth was almost completely suppressed, and median survival increased by >50% relative to control groups. Importantly, mice that had cleared tumors following Bio-HCP@FM-NPs vaccination resisted tumor rechallenge without further immunization, indicating the induction of durable immune memory. Collectively, these findings highlight the power of fusion membrane-derived biomimetic NVs to simultaneously activate innate and adaptive immunity, effectively remodel the TME in lung cancer, and establish long-term protection against recurrence.

The field has also advanced into hybrid or fusion membrane technologies, where different membrane sources are combined to synergize their respective advantages. For example, coating NPs with both tumor and bacterial membranes merge the antigenic breadth of tumor-derived material with the innate immune stimulatory potential of bacterial components. Similarly, fusion of tumor membranes with immune cell membranes, such as those from DCs, creates dual-functional constructs capable of both presenting antigens and delivering strong co-stimulatory signals.^112^

Safety considerations remain at the forefront. While the use of self or patient-derived materials reduces the risk of immunogenicity against irrelevant targets, the potential for triggering autoimmunity or exacerbating inflammation cannot be overlooked because of epitope spreading mechanism.^113^^,^^114^ Incorporating immunostimulatory adjuvants or cytokines within the NP core boosts efficacy, but must be balanced against risks of systemic toxicity or unintended immune activation.^115^

Looking forward, biomimetic and autologous NVs are poised to redefine the landscape of lung cancer immunotherapy. Their unique ability to capture the antigenic complexity of tumors, personalize vaccination to individual patients, and integrate multiple biological functionalities into a single nanosystem marks them as promising next-generation platforms. Ongoing innovations in membrane engineering, such as site-specific modification, controlled fusion, and integration with responsive nanomaterials, are expected to further expand their therapeutic potential. Moreover, combining these vaccines with established immunotherapies, including immune checkpoint inhibitors or adoptive cell transfer, may unlock synergistic effects capable of overcoming some of the most persistent barriers in lung cancer treatment.

### DC-based NVs

DC-based NVs introduce innovative dimensions to cancer immunotherapy by exploiting DC-derived components rather than relying on the direct manipulation of intact cells.^116^ This approach moves beyond conventional *ex vivo* DC vaccines, replacing complex cell culture protocols with nanoscale formulations that capture essential immune-stimulatory functions of DC biology.^117^

One of the most prominent strategies is DC-derived microvesicles (MVs). These extracellular vesicles naturally package surface MHC molecules, co-stimulatory ligands, and cytokines, providing them with a built-in capacity to interface with T cells. When engineered to include TAAs, MVs function as nanoscale surrogates of whole DCs, combining efficient antigen carriage with improved circulation stability and enhanced access to secondary lymphoid tissues. Their nanoscale size confers distinct pharmacokinetic advantages, including better tissue penetration and reduced clearance compared to cell-based formulations. Moreover, MVs derived from genetically modified or activated DCs could be tailored to display heightened levels of stimulatory signals, amplifying their therapeutic performance without the variability like live-cell vaccines.^118^ Zhang et al^94^ reported a polysaccharide-based inhalable NV specifically designed to activate DCs in the lung. Their formulation used dextran derivatives (N,N-dimethylethylenediamine [DMEN] and phenylboronic acid [PBA] modified) as a nanocarrier encapsulating LLC tumor antigens, thus providing both broad-spectrum antigen exposure and intrinsic immunostimulatory properties. *In vitro*, the NV significantly promoted DCs maturation, with ∼2-fold upregulation of CD80/CD86 expression and a 2–3-fold increase in IL-12 and TNF-α release compared to soluble antigens. *In vivo*, intranasal delivery outperformed subcutaneous injection, inducing robust mucosal and systemic immunity. Strikingly, in prophylactic settings, 50% of mice survived beyond 180 days, while all control mice succumbed before day 90. Upon tumor rechallenge, 33% of survivors remained tumor-free, confirming the induction of durable immune memory. Immune profiling revealed enriched CD4⁺ and CD8⁺ T cells, expansion of germinal center B cells, and accumulation of lung-resident memory T cells. Histological analysis confirmed no pulmonary toxicity following repeated dosing. Collectively, this inhalable dextran-based DC-targeting NV demonstrates how mucosal delivery could establish potent and durable protective immunity against lung cancer.

An equally transformative development is the use of mitochondrial DNA (mtDNA) within DC-based NV formulations. MtDNA serves as an intrinsic immunogenic signal capable of triggering the cGAS-STING pathway when delivered to the cytosol, leading to type I interferon secretion and broad proinflammatory programming. Unlike conventional adjuvants, mtDNA combines endogenous origin with potent innate immune activation, enabling a unique synergy when co-delivered with TAAs in DC-based NP systems.^119^ Preclinical lung cancer models demonstrate that such mtDNA-integrated vaccines not only strengthen DC maturation but also expand the breadth of T cell responses, contributing to durable tumor control. This dual mechanism, including antigen presentation and innate pathway stimulation, differentiates mtDNA-based NVs from earlier generations of DC-targeted strategies.^120^

Further refinements include the incorporation of additional molecular cues within these DC-based platforms. Pattern-recognition receptor agonists, cytokine payloads, or ligands for specific DC subsets could be embedded in the nanostructures to fine-tune their functional activity. Adjusting physical characteristics such as vesicle composition, NP elasticity, and cargo release kinetics enables precise control over biodistribution and the balance between immune activation and tolerance.^116^ Shang et al^120^ showed a DC-derived microvesicle (MV_DC_)-based NV platform (cNP_cancer cell_@MV_DC_) engineered to improve antigen presentation and cross-priming in NSCLC. The design involved cationic NPs loaded with mtDNA and tumor lysates, subsequently cloaked with MVs secreted by activated DCs, thereby combining tumor antigens with DC-derived costimulatory cues. *In vitro*, uptake by naïve DCs increased antigen presentation efficiency by over 2-fold and boosted IFN-γ secretion from co-cultured T cells compared with naked lysate controls. *In vivo*, in LLC-bearing mice, treatment induced robust expansion of migratory DCs and NK cells, significantly increased CD8⁺ T cell proliferation, and suppressed tumor growth by nearly 60% relative to controls. Median survival was prolonged by >40%, and vaccinated mice demonstrated enhanced systemic IFN-γ and IL-12 production. Importantly, the platform exhibited cross-cancer efficacy in pancreatic tumors. Notably, in lung cancer models, it achieved substantial tumor suppression and survival benefit without systemic toxicity. This study exemplifies the potential of DC-derived vesicle-based NVs to orchestrate innate and adaptive immunity, offering a promising immunotherapeutic strategy for NSCLC.^50^^,^^120^

Despite their promise, translation of DC-based NVs remains constrained by technical challenges. Reliable methods for the large-scale generation, purification, and quality control of DC-derived vesicles or mtDNA are still evolving, and batch-to-batch variability poses regulatory hurdle. Furthermore, strategies are required to ensure that immunostimulatory potency is preserved during manufacturing and storage. Addressing these barriers will be critical to advancing DC-based NVs into clinical use.

### Fusion-membrane NVs

Fusion-membrane NVs have emerged as an innovative class of immunotherapeutics designed to integrate the strengths of tumor membranes and bacterial membranes within a single nanosystem. This dual-source strategy provides comprehensive antigen coverage together with intrinsic adjuvanticity, offering a powerful means to overcome tumor heterogeneity and the weak immunogenicity.^112^

The central principle of these constructions lies in the physical fusion of two membrane types: tumor membranes harvested from resected tumors or cultured cancer cells, and bacterial membranes engineered to retain immune-stimulatory motifs. Tumor membranes preserve the full repertoire of TAAs and neoantigens in their native conformation, enabling broad polyclonal recognition of diverse tumor clones.^121^ Bacterial membranes supply pathogen-associated molecular patterns (PAMPs) that directly activate pattern recognition receptors, such as TLRs on DCs and other innate effectors.^122^ By embedding TAAs within this adjuvant-rich framework, fusion NVs create a self-contained platform driving both innate activation and adaptive priming without reliance on external adjuvants.^123^

Preclinical studies have demonstrated several unique immunological benefits. Hybrid membrane NPs show efficient biodistribution to secondary lymphoid organs, where they facilitate antigen presentation under strongly proinflammatory conditions. This leads to the proliferation of tumor-specific cytotoxic T cells and support from helper T cells, and simultaneous antibody responses targeting tumor epitopes in certain models.^124^ Notably, in lung cancer, these vaccines have reduced postoperative recurrence, limited metastatic dissemination, and prolonged OS. When administered together with PD-1 inhibition, fusion-membrane formulations display synergistic effects, further enhancing tumor rejection and long-term immune surveillance.^125^ Ling et al^126^ developed an innovative fusion membrane nanoplatform by coating *E. coli* bacterial ghosts with cancer cell membranes from 4T1 tumor cells and further loading them with liposomal paclitaxel (LP@BG@CCM). *In vitro*, LP@BG@CCM exhibited significantly higher cytotoxicity against 4T1 cells compared with free paclitaxel or single-component controls, reflecting the synergistic role of antigen presentation and chemotherapy. In murine lung metastasis models, vaccination with LP@BG@CCM resulted in striking therapeutic benefits: lungs from treated mice appeared nearly normal, lung weight was significantly reduced compared to untreated groups, and histological analysis revealed clear alveolar structures with minimal metastatic foci. Tumor apoptosis was markedly elevated in vaccine-treated animals, as evidenced by terminal deoxynucleotidyl transferase dUTP nick end labeling (TUNEL) staining. Immune profiling additionally demonstrated an expansion of CD4⁺ and CD8⁺ T cells within the spleen, accompanied by increased pulmonary secretion of TNF-α, IFN-γ, and IL-4. This suggests a concurrent activation of the cytotoxic, helper, and humoral components of the immune response. This combinatorial platform effectively prevented metastatic colonization in the lung and reshaped systemic immunity, highlighting fusion membrane NVs as a promising dual-function approach that integrates drug delivery with antigen-specific vaccination in the context of lung cancer.

The structural flexibility of fusion systems also allows for additional engineering refinements. PEGylation extends systemic circulation, while mannose decoration enhances DC targeting. Additionally, the encapsulation of cytokines, such as GM-CSF, within the NP core further augments local immune recruitment.^124^ Such modularity enables tailoring of vaccine performance to specific therapeutic goals, ranging from strong cytotoxic responses to long-lasting memory generation.

Standardizing the preparation of hybrid membranes is technically demanding, as fusion efficiency, antigen density, and bacterial component composition vary across batches.^127^ Large-scale production of patient-specific tumor membranes also presents logistical barriers in clinical translation. Although bacterial motifs are crucial for immune stimulation, excessive activation could lead to systemic inflammation, whereas inadequate incorporation might reduce therapeutic efficacy.^128^ Addressing these issues will require precise control of membrane composition, rigorous quality assurance, and scalable manufacturing pipelines.

### Multi-functional/dual-targeted NVs

Multi-functional, dual-targeted NVs represent an advanced category of cancer immunotherapy platforms by integrating multiple therapeutic components into a single nanosystem. Their design is based on the principle that lung cancer therapy requires simultaneous modulation of distinct biological pathways, including immune activation, suppression of tumor-driven resistance mechanisms, and reprogramming of oncogenic signaling.^129^

One hallmark innovation in this field is the co-encapsulation of tumor antigens together with gene-silencing molecules such as small interfering RNAs (siRNAs). By silencing immunosuppressive mediators, such as PD-L1 or signal transducer and activator of transcription 3 (STAT3), while concurrently delivering TAAs or neoantigens, these NVs orchestrate a dual effect: they restore immune recognition and reduce the inhibitory checkpoints that blunt T cell function.^130^ Experimental examples include nanocarriers loaded with siRNAs targeting PD-L1 and EGFR, which have achieved enhanced vulnerability of tumor cells to cytotoxic attack while stimulating potent antigen-specific T cell responses.^131^, ^132^ Ahn et al^133^ reported on polyethylenimine (PEI)-DNA NPs encoding murine IL-12 under the progression elevated gene 3 (PEG-3) promoter in orthotopic LL/2 and B16F10 metastasis models. Repeated intravenous administration every 3–4 days significantly prolonged survival relative to controls and recombinant cytokine therapy. Importantly, plasmids with reduced CpG content retained efficacy but induced lower acute cytokine release, confirming that the therapeutic effect was IL-12-driven rather than TLR9-mediated. These findings illustrate how multifunctional NVs integrate cytokine gene delivery with antigen stimulation to convert “cold” tumors into inflamed, T cell-responsive lesions.

A multifunctional NV platform was engineered utilizing poly(ethylene glycol)-poly(ε-caprolactone) copolymers (PEG-PCL) NPs encapsulating verteporfin, a Food and Drug Administration (FDA)-approved photosensitizer. This platform was designed with matrix metalloproteinase (MMP)-responsive linkers to enable tumor-specific release. When combined with hypofractionated radiotherapy (RT) (2 Gy × 3 fractions), this formulation induced ICD comparable to conventional high-dose RT (8 Gy), but with reduced toxicity. In LLC models, treated mice displayed significant tumor growth suppression and evidence of systemic immune activation: DC maturation was enhanced, serum IFN-γ and TNF-α levels were elevated, and abscopal effects were observed, with regression of untreated distant lesions. Flow cytometry revealed increased infiltration of CD8⁺ effector T cells and reduced Treg and MDSC populations within tumors. Survival analyses confirmed a robust benefit, with median survival nearly doubling compared to RT alone. Importantly, histological evaluation showed minimal off-target toxicity in normal lung tissue, confirming the safety of the combined strategy. These findings underscore how multifunctional NVs coupling radiosensitization with *in situ* vaccination can amplify antitumor immunity and overcome resistance mechanisms in lung cancer.^134^

Another distinctive approach involves integrating TAAs with molecular immune activators or regulatory cargos in one NP. This includes TLR ligands, STING agonists, cytokines such as GM-CSF, or even low-dose chemotherapeutics capable of inducing ICD. The synchronized release of these agents ensures that antigen presentation is coupled with innate immune activation, producing a concerted signal that favors durable adaptive responses. In certain formulations, NPs are engineered to react to tumor-associated stimuli, including acidic pH, redox gradients, or enzymatic activity. This design facilitates spatiotemporally controlled release of therapeutic agents while minimizing systemic toxicity.^22^^,^^135^^,^^136^

Preclinical investigations in lung cancer and other solid tumors have demonstrated that multi-component NVs are capable of broad immunological reprogramming. They enhance DC priming, promote the expansion of polyfunctional T cells, diminish regulatory populations such as Tregs and MDSCs, and suppress metastatic spread. Importantly, the induction of ICD by certain formulations further extends the antigenic repertoire presented to the immune system, reinforcing vaccine efficacy. When used in combination with immune checkpoint inhibitors, these NVs have shown additive or synergistic effects, resulting in superior tumor regression and long-term survival.^137^

Achieving precise control over stability, bioavailability, and release kinetics of diverse cargos is technically demanding. Risks of off-target gene silencing or unwanted immune activation must be mitigated, and scalable manufacturing processes for such complex nanostructures are still under development. Furthermore, identifying biomarkers that guide the rational pairing of antigens with regulatory molecules will be crucial for effective patient stratification.^138^^,^^139^

### Induction of ICD, PANoptosis, and immune memory

The most transformative innovation in NV for lung cancer is the purposeful induction of ICD, which converts dying tumor cells into powerful adjuvants that extend the immune response beyond conventional antigen delivery. ICD is mechanistically distinct from apoptosis or necrosis, being characterized by the emission of damage-associated molecular patterns (DAMPs) such as calreticulin (ectopic calreticulin, ecto-CRT), extracellular adenosine triphosphate (ATP), and high mobility group box 1 (HMGB1).^140^ These signals not only mark tumor cells as “immunologically visible”, but also provide essential signals for DCs recruitment, phagocytosis, and cross-presentation of tumor antigens.^141^ For example, calreticulin exposure functions as an “eat-me” signal for DCs, ATP acts as a chemoattractant through purinergic receptor signaling, and HMGB1 promotes TLR-mediated DCs activation.^142^ The combined release of these molecules ensures that both endogenous tumor antigens and NVs-delivered antigens are processed in a strongly immunostimulatory context.

NV engineering has leveraged ICD via multiple strategies. Some platforms encapsulate chemotherapeutic agents such as doxorubicin or oxaliplatin within NPs to induce ICD directly inside tumors. Others employ photodynamic or photothermal nanomaterials, which generate reactive oxygen species or hyperthermia upon light irradiation, triggering regulated tumor cell death while simultaneously releasing tumor antigens.^21^ Additionally, oncolytic viral mimetics or engineered peptides have been incorporated into nanocarriers to promote ICD and enhance antigen exposure. Beyond direct inducers of cell death, NVs increasingly combine immune agonists (STING or TLR ligands) with tumor antigens to couple the release of DAMPs with the activation of innate immunity, thereby reinforcing the maturation of DCs and the priming of effector T cells.^143^

PANoptosis is a programmed cell death pathway characterized by inflammation, integrating pyroptosis, apoptosis, and necroptosis through the assembly of PANoptosomes, a multiprotein complex. Sensors such as Z-DNA-binding protein 1 (ZBP1), absent in melanoma 2 (AIM2), NOD-like receptor family pyrin domain containing 3 (NLRP3), and receptor-interacting serine/threonine-protein kinase 1 (RIPK1), which respond to various stimuli, including infections and cellular stress, are involved in this process. In cancer, particularly lung cancer, tumor resistance to cell death promotes progression.^144^^,^^145^ However, activation of PANoptosis can enhance ICD, promote the release of DAMPs, and remodel the tumor immune microenvironment (TIME) by increasing T cell infiltration and macrophage polarization. Of note, higher expression of PANoptosis-related genes is correlated with improved prognosis, longer disease-free survival, reduced metastasis, and increased immune cell infiltration.^146^ Unlike classical ICD, which is typically linked to isolated modes of cell death, PANoptosis produces a broader inflammatory milieu and more comprehensive elimination of tumor cells. NVs designed to initiate PANoptosis have shown the ability to release diverse antigenic epitopes, intensify cytokine production, and enhance immune infiltration within the lung cancer TME. In preclinical lung cancer models, PANoptosis-inducing NVs have also been applied to reprogram TAMs from immunosuppressive to proinflammatory phenotype, dismantling immunosuppressive barriers while simultaneously amplifying T cell responses.^147^ This dual function, including tumor clearance and stromal reprogramming, suggests PANoptosis-based strategies may overcome the mechanisms of resistance. Chung et al^148^ used viral NP (VNP) vaccines based on bacteriophage Qβ virus-like particles (VLPs) and cowpea mosaic virus (CPMV) to target S100A9 in pre-metastatic lung niches. In B16F10 models, Qβ-S100A9 vaccination resulted in 78% reduction in lung tumor nodules compared to the Qβ control, and CPMV-S100A9 resulted in 91% reduction compared to the CPMV control. In 4T1 and TNBC metastatic model, the Qβ-S100A9 vaccine resulted in a 62% reduction in lung tumor nodules compared to the Qβ control. Survival improved by ∼267–400% compared with controls, correlating with suppression of lung S100A8/9 protein surges (13,767 ng/mL in naïve *vs*. 360.3 ng/mL in vaccinated mice). These data highlight the ability of ICD-linked NVs to block metastatic conditioning and establish long-term protective immunity.

The clinical relevance of ICD and PANoptosis lies in their capacity to support long-term immunological memory. Antigens released during these forms of cell death encompass a broad repertoire, including neoantigens and variants produced during tumor stress, thereby fostering the development of tumor-specific central memory (Tcm), effector memory (Tem), and Trm cells.^22^^,^^142^ By sustaining both local and systemic memory responses, ICD- and PANoptosis-based NVs ensure extended protection well beyond initial tumor clearance.

An innovative *in situ* vaccination strategy leveraged PEGylated gold NPs (AuNPs) for photothermal tumor ablation in combination with R848-functionalized nanocarriers to amplify antigen capture and immune activation. Upon the second near-infrared (NIR-II) laser irradiation, localized hyperthermia induced ICD, releasing a broad spectrum of TAAs. Concurrently, R848-decorated vesicles captured these antigens and migrated to draining lymph nodes, where they promoted potent DCs maturation and T cell priming. In lung metastasis models, treated mice exhibited robust CD8⁺ T cell expansion, with tumor-specific IFN-γ⁺ T cells increased 3-fold over controls. Therapeutic outcomes were equally striking: pulmonary metastatic nodules were significantly reduced, secondary tumor formation was suppressed, and survival was markedly extended, with median survival exceeding 60 days compared to ∼30 days in untreated mice. Histological analysis confirmed dense infiltration of CD8⁺ T cells within metastatic lesions, while serum cytokine profiles showed elevated IL-12 and type I interferons, consistent with durable systemic immunity. These data highlight the capacity of NIR-II photothermal NVs to couple localized tumor ablation with systemic immune priming, a powerful approach for metastatic lung cancer.^149^

Another key dimension is the synergy with checkpoint blockade therapy. Tumors undergoing ICD and PANoptosis exhibit an increased presence of antigen presentation machinery and proinflammatory cytokines, thereby reinstating their sensitivity to inhibitors targeting PD-1/PD-L1 or cytotoxic T-lymphocyte-associated protein 4 (CTLA-4) pathways.^21^ Preclinical studies demonstrate that NVs inducing ICD markedly enhance responsiveness to checkpoint inhibitors, leading to durable tumor regression and prolonged survival.^150^ This combination approach is especially valuable in lung cancer, where immune escape and heterogeneity frequently limit the efficacy of single-modality therapies.

## Translational challenges and clinical considerations

**Fig. 3:**
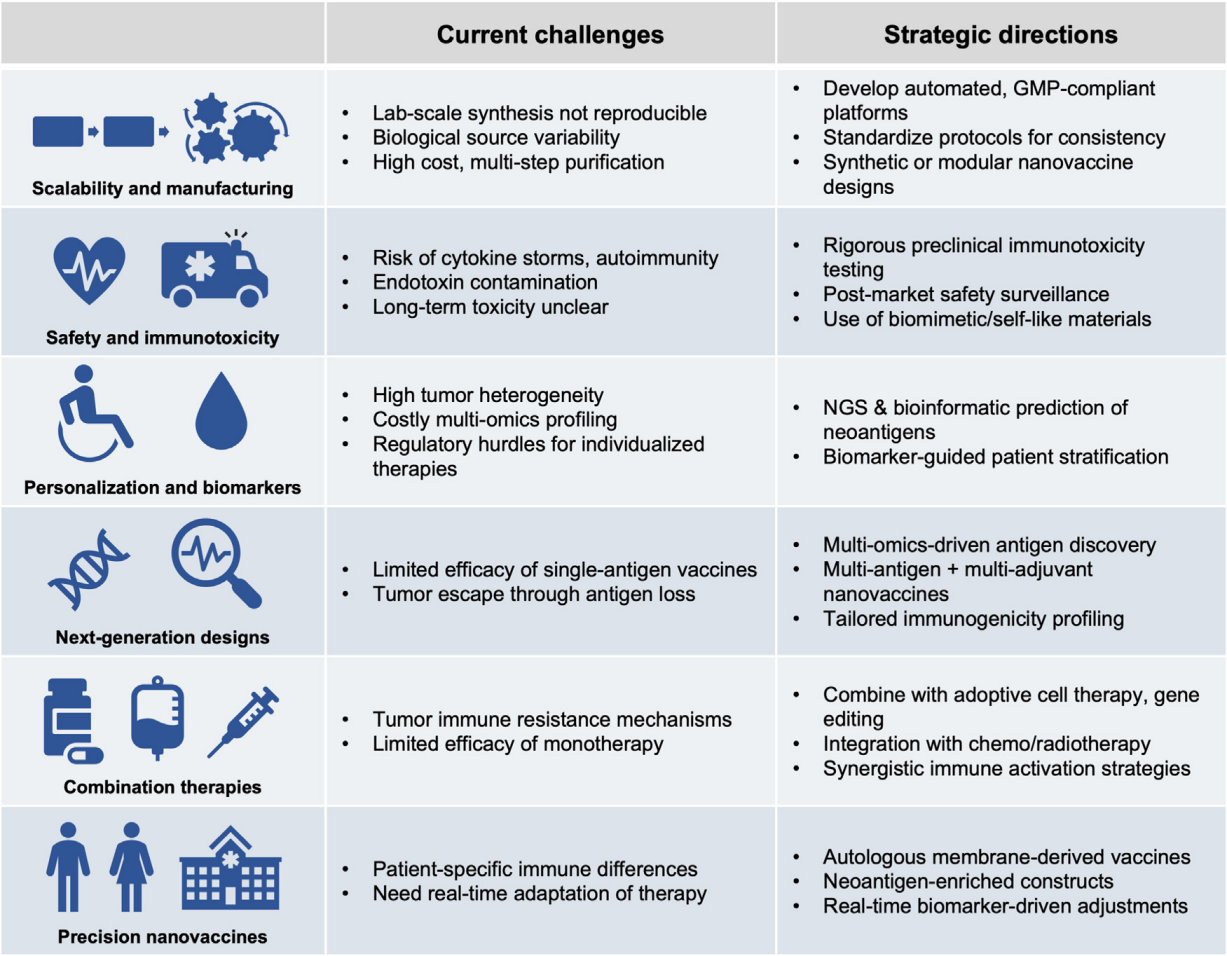
Translational challenges and future perspectives. Major aspects include scalability and manufacturing, safety and immunotoxicity, personalization and biomarker integration, next-generation vaccine designs, combination therapeutic strategies, and precision NVs. These strategies outline a roadmap toward safe, scalable, and personalized NV platforms for clinical translation in lung cancer immunotherapy. GMP, Good manufacturing practice; NGS, Next generation sequencing; NV, Nanovaccine.

Despite the impressive success of NV platforms in preclinical models of lung cancer, the transition from bench to bedside presents a distinct set of challenges. Bridging the gap between experimental efficacy and real-world clinical application requires careful consideration of multiple factors, including scalability, safety, manufacturing consistency, regulatory pathways, and patient-specific variables (Fig. 3).^151^

### Scalability and manufacturing

A critical challenge in translating NV platforms for lung cancer from laboratory discovery to clinical application lies in the scalability and reproducibility of manufacturing processes. While preclinical successes often rely on small-batch, highly controlled synthesis, the production of NVs at clinical or commercial scale requires robust, standardized, and cost-effective methods that maintain product quality and consistency across multiple batches.^147^ This is particularly pertinent considering the complexity inherent in numerous NV constructs, such as those incorporating whole cell membranes, recombinant proteins, or fusion-membrane components. These constructions frequently necessitate multi-step purification processes, precise antigen quantification, and the incorporation of adjuvants or immune modulators within the NP matrix.^152^

One major limitation is the variability introduced by biological source materials, such as autologous tumor cell membranes or patient-derived proteins, which differ not only between individuals but also between tumor samples from the same patient over time. Ensuring lot-to-lot consistency, purity, and stability of such biologically derived materials remains an unresolved obstacle for large-scale production.^153^ Moreover, the technical requirements for isolating, purifying, and validating these complex components add significant time and cost to the manufacturing pipeline.

Advancements in automated synthesis, high-throughput purification, and quality control technologies have enhanced the scalability and reproducibility of NVs.^154^ Nonetheless, the need for stringent control over NP size, surface charge, antigen loading, and release kinetics continues to pose technical challenges as the process is scaled up from research-grade to clinical- or Good Manufacturing Practice (GMP)-grade production.^155^

Regulatory standards for nanomedicine manufacturing are still evolving, and many NV platforms must address the specific requirements for sterility, endotoxin removal, long-term storage, and *in vivo* stability.^153^ The absence of standardized industry protocols for certain advanced platforms, notably biomimetic and fusion-membrane systems, exacerbates the challenges associated with harmonizing production practices.^156^ The development of scalable, reproducible, and economically viable manufacturing solutions is thus a central priority for the clinical translation of NV immunotherapies.^157^

Ultimately, close collaboration among researchers, biomanufacturing specialists, and regulatory agencies will be required to overcome these challenges and ensure that innovative NV technologies could be reliably produced and delivered at a scale suitable for broad clinical application in lung cancer.

### Safety and immunotoxicity

The safety and immunotoxicity profile of NV platforms is a critical determinant of their potential for successful clinical translation in lung cancer therapy.^158^^,^^159^ While the vast majority of preclinical studies report generally favorable safety outcomes, the unique physicochemical properties and biological complexity of NVs demand rigorous assessment and long-term surveillance.^160^

In studies utilizing animal models, the majority of NV formulations, encompassing mRNA-based, protein/peptide, biomimetic, and fusion-membrane systems, have exhibited minimal acute toxicity. These formulations have shown no significant adverse effects on body weight, organ histology, or serum biochemistry.^104^ Biomimetic and autologous membrane-derived NVs, in particular, benefit from their “self-like” characteristics, which often result in reduced immunogenicity against non-target tissues and minimal off-target effects.^161^ Nonetheless, the inclusion of immune-stimulating adjuvants, bacterial membrane components, or fusion-membrane designs necessitates caution, as excessive or uncontrolled activation of the immune system could theoretically provoke local or systemic inflammation, cytokine release syndrome, or autoimmunity.^162^

Another important consideration is unintended immune suppression or immunopathology. Certain NV components, such as nucleic acid-based adjuvants or repeated administration of strong immune agonists, may have the potential to induce immune tolerance, T cell exhaustion, or disrupt the balance of regulatory and effector cell populations.^62^ The presence of endotoxins or contaminants in biologically derived materials also poses a potential hazard, requiring stringent purification and quality control during manufacturing.^163^

Long-term immunotoxicity data are still limited, both in animal models and especially in humans. While most studies report rapid clearance and favorable biocompatibility of NV platforms, subtle effects on immune homeostasis, hypersensitivity, or delayed adverse events cannot be ruled out, particularly with repeated dosing or chronic administration. Additionally, the potential for NP accumulation in specific organs (such as the liver, spleen, or lung) and the effects on tissue-resident immune cells warrant further investigation.^164^

Regulatory authorities are placing heightened emphasis on the safety profile of nanomedicines, underscoring the necessity for extensive preclinical evaluation. This includes conducting biodistribution studies, immunopathological assessments, and sensitive immunotoxicity assays before proceeding to clinical application.^157^ The evolving landscape of NV development thus requires a proactive approach to safety assessment, with transparent reporting, post-market surveillance, and a commitment to understanding both the benefits and risks associated with advanced NP-based immunotherapies.^158^

In summary, while preclinical findings are encouraging and most NVs appear to be well-tolerated, the complexity of these platforms calls for ongoing vigilance and a cautious, data-driven approach as they advance toward clinical use in lung cancer.

### Personalization and biomarker-guided approaches

Personalization is rapidly becoming a central theme in the development and application of NV immunotherapies for lung cancer. Given the remarkable heterogeneity of tumor antigens, mutational profiles, and immune landscapes across individual patients, a “one-size-fits-all” strategy is increasingly recognized as insufficient for achieving durable and effective anti-tumor responses.^165^

A major advance is the identification and exploitation of patient-unique tumor neoantigens through next-generation sequencing (NGS) and bioinformatic prediction.^166^ By integrating genomic, transcriptomic, and proteomic data, researchers select the most immunogenic and relevant epitopes for personalized NV constructs, whether as mRNA, peptides, or whole tumor lysate.^167^ Such individualized vaccines have demonstrated the ability, at least in preclinical and early clinical models, to induce robust, polyclonal T cell responses and overcome the limitations of tumor heterogeneity and immune escape.^62^

Biomarker-guided approaches further enhance personalization by enabling the stratification of patients according to likely benefit from specific NV platforms. Predictive biomarkers, including TMB, HLA type, pre-existing immune infiltration, expression of immune checkpoints, and the abundance of specific myeloid cell populations, are increasingly employed to inform patient selection, vaccine design, and the selection of combination therapies. TMB is a prominent candidate, as a high TMB is associated with increased neoantigen load, which enhances vaccine-induced T cell responses in NSCLC, particularly in tumors related to smoking.^168^ High TMB indicates improved outcomes with neoantigen-targeted therapies.^169^ The integration of these biomarkers maximizes therapeutic efficacy and minimizes the risk of unnecessary toxicity or ineffective intervention.^170^

Ongoing research is also focused on the development of real-time, dynamic biomarkers to monitor treatment response and adapt therapeutic strategies. These may include circulating tumor DNA (ctDNA), immune cell phenotyping, cytokine profiles, or even advanced imaging techniques to track immune cell infiltration and tumor regression. By closely aligning vaccine design and clinical decision-making with such biomarker data, the potential for precision immunotherapy in lung cancer is greatly expanded.^171^

Addressing the complexity and cost of comprehensive tumor profiling, the necessity for expedited production of personalized vaccines, and the regulatory challenges associated with individualized therapies necessitates the development of innovative solutions. Manufacturing of personalized vaccines is also very costly. Nevertheless, the trajectory of the field clearly points toward a future in which NVs based immunotherapy for lung cancer is not only broadly effective but also precisely tailored to the unique molecular and immunological characteristics of each patient.

## Future perspectives

As NV research in lung cancer continues to advance, the field stands at a pivotal crossroads between scientific innovation and clinical translation. The lessons learned from preclinical successes, combined with an evolving understanding of tumor immunology and emerging technological breakthroughs, are shaping a new generation of strategies aimed at overcoming current limitations. Looking ahead, future perspectives center on optimizing NV design, personalizing therapeutic approaches, and integrating novel combinations to unlock deeper and more durable anti-tumor immunity.^172^

### Next-generation NV designs

Designing the next generation of NVs for lung cancer is increasingly shaped by lessons from experimental studies showing the complex and heterogeneous nature of tumor antigens and the immune landscape. In the reviewed body of research, it has become clear that the response to simple, single-antigen vaccines is often limited by antigen loss, tumor evolution, and variability in how the immune system recognizes different tumors. This recognition has prompted a major shift toward integrating a broader spectrum of tumor-derived materials in vaccine formulations.^173^ Multi-omics approaches, including the integration of genomic, transcriptomic, and proteomic analyses, are increasingly proposed as essential tools for the rational selection of immunogenic targets. By comprehensively profiling tumor mutations, gene expression patterns, and antigenic landscapes, researchers could identify patient-specific neoantigens, immune evasion pathways, and biomarkers predictive of vaccine responsiveness. This deep profiling enables the development of NVs that are precisely tailored to the molecular and immunological features of each patient’s tumor, thereby increasing the likelihood of durable and effective anti-tumor immunity.^174^ In addition to target selection, the inclusion of multiple antigens within a single NV formulation is repeatedly highlighted as a strategy to enhance immune coverage and reduce the risk of tumor escape through antigen loss or heterogeneity. Animal studies demonstrate that NVs based on whole tumor lysates, cell membrane extracts, or cocktails of peptide and protein antigens can elicit broader, polyclonal T cell responses and confer protection against both primary and metastatic disease.^174^

The reviewed literature also underscores the potential of multi-adjuvant systems, in which more than one innate immune stimulator is incorporated into the NV platform. For instance, the simultaneous delivery of TLR agonists (e.g., CpG or poly(I:C)), STING pathway activators, or cytokines such as GM-CSF within a single NP synergistically augments DC maturation, antigen presentation, and T cell priming. These combinatorial adjuvant strategies have shown superior results in animal models compared to single-adjuvant formulations, leading to more potent and sustained anti-tumor responses.^175^

Taken together, these avenues indicate that forthcoming NVs for lung cancer are poised to be highly personalized, multi-modal constructs. They will integrate insights from multi-omics profiling, deliver multiple tumor antigens, and leverage the synergistic effects of combined adjuvants. The convergence of these advances, already visible in the latest experimental designs and proposals within the reviewed articles, sets the stage for more robust, flexible, and clinically relevant NV platforms in the fight against lung cancer.

### Potential for combination with other modalities

The future direction of NV development for lung cancer is increasingly defined by the potential for strategic combination with other therapeutic modalities. As several studies in this review highlight, the complexity of lung cancer biology and its mechanisms of immune resistance often demand a multifaceted treatment approach. Of note, the incorporation of NVs into multimodal treatment for lung cancer necessitates meticulous attention to the timing—whether it be neoadjuvant (prior to surgery), adjuvant (following surgery), or maintenance phases—to enhance immune priming while reducing disruption to conventional therapies.^176^^,^^177^

Preclinical investigations have demonstrated that the therapeutic effects of NVs can be significantly enhanced when integrated with other immunotherapy, cell-based therapies, or even gene-modulating strategies.^174^ Bispecific antibodies constitute a novel class of combinatorial partners. These agents facilitate the redirection of endogenous T cells towards tumor cells; however, they frequently encounter challenges in solid tumors, primarily due to inadequate T-cell infiltration and the absence of lasting memory responses.^178^

A promising approach, as demonstrated in animal models, involves the integration of NV immunization with adoptive cell therapies, including the transfer of tumor-infiltrating lymphocytes or engineered T cells such as chimeric antigen receptor (CAR)-T cells, gene-edited T cells and armored T cells. In these contexts, NVs have the potential to prime the host immune system, enhance the expansion of antigen-specific T cell populations, and modify the TME to support the infiltration and sustained presence of adoptively transferred cells.^179^

Similarly, several reviewed articles discuss the potential integration of NV approaches with gene therapy. For example, co-delivery of NV formulations alongside vectors or NPs carrying gene-silencing elements, such as siRNA or clustered regularly interspaced short palindromic repeats (CRISPR)-based tools targeting immunosuppressive pathways, augments anti-tumor responses by simultaneously reducing immune escape mechanisms. Some NV platforms incorporate siRNA or other genetic payloads designed to inhibit checkpoint molecules or reprogram the TME, thereby potentiating the effects of immune activation.^164^^,^^179^

Additional combinatorial approaches under investigation involve the integration of NVs with established treatments, such as chemotherapy and radiotherapy. Certain chemotherapeutic agents and radiotherapy induce ICD, thereby enhancing the release of tumor antigens and DAMPs. When administered at an optimal time, NVs exploit this period to significantly enhance DCs activation and T cell priming.^21^

Collectively, these avenues of research, as evidenced by numerous animal studies and theoretical frameworks within the reviewed literature, highlight the significant potential of NV platforms to serve as a pivotal element in multi-modal cancer immunotherapy. The strategic integration of NVs with cell therapy, gene therapy, and established or emerging cancer treatments is expected to open new avenues for overcoming resistance, enhancing efficacy, and personalizing lung cancer care in the future.

### Outlook for personalized NVs in lung cancer

Personalized and precise NV strategies are rapidly gaining attention as a transformative direction in lung cancer immunotherapy. This shift is driven by the recognition that tumor heterogeneity and individual immune differences critically influence treatment outcomes. Recent studies indicate that platforms such as autologous membrane-derived NVs and neoantigen-enriched formulations illustrate the potential for tailoring vaccine design to the unique genetic, proteomic, and immunologic characteristics of individual tumors, thereby enhancing both the magnitude and specificity of anti-tumor responses.^158^

Experimental models utilizing patient-derived or individualized tumor materials have shown that such approaches broaden the repertoire of presented antigens, support polyclonal T cell immunity, and reduce the likelihood of immune escape. For instance, antigen-enriched tumor cell membrane (AECM) NVs, combined with a STING-activating adjuvant, induced robust poly-neoepitopic CD8⁺ T cell responses and significant tumor regression in murine models.^108^

Preliminary clinical findings, albeit limited, indicate that vaccines customized to a patient’s unique neoantigen profile, particularly when used in conjunction with checkpoint inhibitors, have the potential to produce more sustained responses, even in cases exhibiting resistance. Furthermore, several studies highlight the importance of real-time biomarker monitoring, such as tracking T cell responses, cytokine levels, or TMB, to adapt vaccine composition, adjuvant selection, and dosing dynamically in accordance with tumor and host immunity evolution.^180^

The integration of NGS and bioinformatic antigen prediction enables precise identification of tumor-specific neoantigens and supports patient stratification for NV immunotherapy.^181^

Collectively, these experimental and translational insights strongly support the promise of personalized and precise NVs in lung cancer. As the field advances, developing scalable, adaptable, and patient-centered vaccine technologies is expected to shift the paradigm from generic immunization to truly individualized cancer therapy.

## Conclusion

The field of NV immunotherapy for lung cancer has witnessed remarkable advances in recent years, driven by innovations in vaccine design, delivery systems, and deeper understanding of tumor immunology. The collective findings from experimental studies underscore the critical role of precise antigen selection, robust DCs activation, and effective modulation of the TME in shaping anti-tumor immune responses. Various NV platforms, encompassing mRNA-based formulations, protein/peptide carriers, biomimetic and autologous membrane constructs, as well as multifunctional hybrid systems, have demonstrated the ability to elicit robust, polyclonal T cell responses. These platforms have been shown to effectively inhibit primary tumor growth and metastatic spread. Furthermore, in numerous preclinical models, they have been observed to generate durable immune memory, which is capable of preventing tumor recurrence.

Despite these promising results, the path to clinical translation is shaped by a range of challenges, from scalability and reproducibility in manufacturing to the need for comprehensive safety evaluation and regulatory oversight. The intrinsic heterogeneity of lung cancer, along with the complexity of the immune landscape, highlights the necessity for personalized and biomarker-guided approaches. Integrating multi-omics data, refining multi-antigen and multi-adjuvant strategies, and combining NVs with other treatment modalities, including checkpoint inhibitors, cell therapies, and gene modulation, offer exciting opportunities to further improve outcomes.

Animal studies have provided strong proof-of-concept evidence for the therapeutic potential of NVs, demonstrating not only tumor inhibition and remodeling of the TME, but also the feasibility of combination and personalized approaches. Early clinical experiences, though still limited, suggest that patient-tailored NVs could be transformative, particularly for cases refractory to existing therapies.

Looking forward, the continued evolution of NV research depends on multidisciplinary collaboration, advances in bioinformatics and sequencing technologies, and close integration of laboratory discoveries with clinical practice. The ultimate goal is to deliver highly effective, durable, and safe immunotherapies that can be tailored to the unique biology of each patient and tumor. With sustained innovation and rigorous validation, NV-based immunotherapy has the potential to significantly alter the landscape of lung cancer treatment, offering hope for improved survival and long-term disease control.

## CRediT authorship contribution statement

**Hamed Hosseinalizadeh:** Writing – original draft. **Fujing Ge:** Writing – original draft. **Mohsen Davari:** Writing – review & editing. **Xiaohong Liu:** Writing – review & editing. **Lei Tian:** Writing – review & editing. **Jianhua Yu:** Writing – review & editing, Writing – original draft, Supervision, Conceptualization.

## Declaration of competing interest

The authors declare that they have no known competing financial interest or personal relationships that could have appeared to influence the work reported in this paper.
